# *Trypanosoma cruzi* reprograms mitochondrial metabolism within the anterior midgut of its vector *Rhodnius prolixus* during the early stages of infection

**DOI:** 10.1186/s13071-024-06415-1

**Published:** 2024-09-06

**Authors:** Radouane Ouali, Larissa Rezende Vieira, Didier Salmon, Sabrina Bousbata

**Affiliations:** 1 Laboratory of Vector-Pathogen Biology, Proteomic Platform, Department of Molecular Biology, Université Libre de Bruxelles, 6041 Gosselies, Belgium; 2 Institute of Medical Biochemistry Leopoldo de Meis, Centro de Ciências e da Saúde, Federal University of Rio de Janeiro, Rio de Janeiro, 21941-902 Brazil

**Keywords:** Chagas disease, Mitochondria, Quantitative proteomics, Host–parasite interaction

## Abstract

**Background:**

*Trypanosoma cruzi* is transmitted to humans by hematophagous bugs belonging to the Triatominae subfamily. Its intra-vectorial cycle is complex and occurs exclusively in the insect's midgut. Dissecting the elements involved in the cross-talk between the parasite and its vector within the digestive tract should provide novel targets for interrupting the parasitic life cycle and affecting vectorial competence. These interactions are shaped by the strategies that parasites use to infect and exploit their hosts, and the host's responses that are designed to detect and eliminate parasites. The objective of the current study is to characterize the impact of *T. cruzi* establishment within its vector on the dynamics of its midgut.

**Methods:**

In this study, we evaluated the impact of *T. cruzi* infection on protein expression within the anterior midgut of the model insect *Rhodnius prolixus* at 6 and 24 h post-infection (hpi) using high-throughput quantitative proteomics.

**Results:**

Shortly after its ingestion, the parasite modulates the proteome of the digestive epithelium by upregulating 218 proteins and negatively affecting the expression of 11 proteins involved in a wide array of cellular functions, many of which are pivotal due to their instrumental roles in cellular metabolism and homeostasis. This swift response underscores the intricate manipulation of the vector's cellular machinery by the parasite. Moreover, a more in-depth analysis of proteins immediately induced by the parasite reveals a pronounced predominance of mitochondrial proteins, thereby altering the sub-proteomic landscape of this organelle. This includes various complexes of the respiratory chain involved in ATP generation. In addition to mitochondrial metabolic dysregulation, a significant number of detoxifying proteins, such as antioxidant enzymes and P450 cytochromes, were immediately induced by the parasite, highlighting a stress response.

**Conclusions:**

This study is the first to illustrate the response of the digestive epithelium upon contact with *T. cruzi*, as well as the alteration of mitochondrial sub-proteome by the parasite. This manipulation of the vector's physiology is attributable to the cascade activation of a signaling pathway by the parasite. Understanding the elements of this response, as well as its triggers, could be the foundation for innovative strategies to control the transmission of American trypanosomiasis, such as the development of targeted interventions aimed at disrupting parasite proliferation and transmission within the triatomine vector.

**Graphical Abstract:**

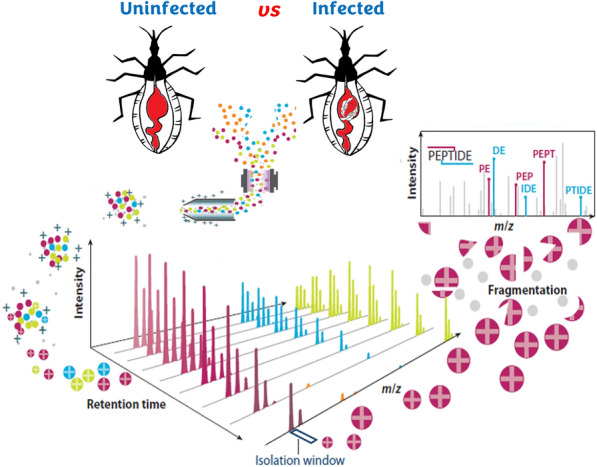

**Supplementary Information:**

The online version contains supplementary material available at 10.1186/s13071-024-06415-1.

## Background

*Trypanosoma cruzi* is the etiological agent of American trypanosomiasis, commonly known as Chagas disease, a potentially lethal parasitic infection afflicting approximately 6 million individuals globally and resulting in the death of no fewer than 12,000 annually [1]. *Trypanosoma cruzi* is principally transmitted to humans by triatomine bugs, among which *Rhodnius prolixus*, *Triatoma infestans*, *Panstrongylus megistus*, and *Triatoma dimidiata* are considered the most important vector species. [2]. These hematophagous insects acquire the parasite when feeding on the blood of infected mammals. Upon ingestion, the parasite develops exclusively within the vector’s digestive system through different stages of its life cycle, before reaching the infective form, which is excreted with the feces and infects the vertebrate host through the biting site [3–5].

During the first hours of the intra-vectorial cycle, the parasite undergoes significant mortality within the anterior midgut (AM) [6, 7]. This severe reduction in parasite number could be caused by digestion-related factors secreted in response to blood ingestion in this organ [7–9], or by the immune response mounted by the insect in order to control potential pathogens contained in the ingested meal [10–12]. Indeed, we have recently demonstrated that *T. cruzi* infection triggers a systemic immune response mediated by the immediate overexpression of immune proteins such as defensins, and the induction of the phenol oxidase response in the hemolymph at 24 h post-infection (hpi) [13]. Thereafter, the surviving parasites migrate to the posterior midgut (PM), where they differentiate into replicative epimastigotes [7]. These forms adhere to the perimicrovillar membranes (PMM) of the intestinal cells and actively multiply [4, 8, 14]. After colonizing the rectum and adhering to the rectal walls, the parasites further differentiate into metacyclic infective trypomastigotes [15, 16].

The digestive tract of triatomines is the interface for triatomine–trypanosome interactions, and vectorial competence toward *T. cruzi* transmission is closely linked to its homeostasis [17–19].

Several investigations have revealed that *T. cruzi* subtly affects the behavior, development, physiology, and life history traits of nymphs and adult triatomines (reviewed in 18). Indeed, infected nymphs show reduced locomotion and prolonged development and molting times. They also ingest more blood and have reduced starving ability compared to uninfected nymphs. Additionally, infection slightly reduces both egg-laying and hatching rates. [18–21]. Despite these observations, very little is known about the molecular impact of the parasite on triatomines’ digestive tract dynamic. However, it has been demonstrated that *T. cruzi* infection alters the expression levels of transcripts in both the midgut and salivary glands of *T. infestans* [22] and *Rhodnius neglectus* [23]. Hence, we posit that examination of *T. cruzi* establishment within its vector on the dynamic of its midgut can unveil fundamental insights into vector–pathogen cross-talk during the parasite’s developmental cycle. Consequently, this understanding could lead to novel control strategies by targeting promising protein candidates crucial for the establishment of the parasite, ultimately impeding transmission to the vertebrate host.

In pursuing this objective, we applied comparative proteomics to evaluate the impact of *T. cruzi* infection on protein expression in the midgut of the model insect *R. prolixus*, aiming to elucidate the metabolic changes induced by the parasite. These data showed an important modulation of mitochondrial functions and additional diverse functions likely related to mitochondria such as redox regulation and detoxification. These critical insights are pivotal in deepening our comprehension of the parasitic life cycle within the vector, illuminating previous observations regarding the parasite's effect on the vector.

## Methods

### Parasites

*Trypanosoma cruzi* Dm28c clone epimastigotes (Carabobo, Venezuela) were grown in liver infusion tryptose (LIT) culture medium containing 10% heat-inactivated fetal bovine serum at 28 °C [24]. Epimastigote forms used in this study were obtained during the log growth phase. The parasites were counted using a Neubauer counting chamber.

### Insects and midgut tissue preparation

*Rhodnius prolixus* adult females were maintained at a stable temperature of 28 °C and 60–80% humidity, in controlled environment incubators, at a female/male ratio of 4:1, with a photoperiod of 12:12 h light/dark (Federal University of Rio de Janeiro, Rio de Janeiro, Brazil). Insects were fed through a latex artificial membrane feeding apparatus with normal heparinized (2.5 units/ml) rabbit blood. Infected insects were artificially fed with *T. cruzi* epimastigote-infected rabbit blood (heat-inactivated), containing 10^7^ epimastigotes/ml. Insects were fed 3 weeks after the first blood meal, and only insects showing successful feeding were selected. Midguts were dissected from both uninfected and *T. cruzi*-infected females at 6 and 24 h post-feeding. The AM were recovered separately from the other gut tissues. AM were rigorously washed with cold phosphate-buffered saline (PBS) 0.01 M and stored in STE buffer (0.1 M Tris, 0.05 M NaCl, 0.05 M ethylenediaminetetraacetic acid [EDTA], pH 7.4) with 1% v/v proteases inhibitor cocktail (Sigma-Aldrich Co., St. Louis, MO, USA) at −80 °C until use. Each biological replicate in this study represents five pooled tissues, with a total of four biological replicates per condition. In order to obtain the AM protein extracts, the tissues underwent a freeze–thaw process at −80 °C, followed by three cycles of ultrasonic water bath sonication for 5 min each. Subsequently, the samples were centrifuged at 13,000×*g* for 15 min at 4 °C. The supernatant was collected, and the extracted proteins were quantified using the Pierce 660 nm Protein Assay (Thermo Scientific Inc., Rockford, IL, USA). The protein extracts were then subjected to verification of their integrity through profile analysis by sodium dodecyl sulfate–polyacrylamide gel electrophoresis (SDS-PAGE) on a tris-tricine 12% gel.

### Sample preparation prior to liquid chromatography–tandem mass spectrometry (LC–MS/MS)

For sample preparation, we used the iST-BCT kit for bottom-up proteomic sample preparation according to the manufacturer’s instructions (PreOmics GmbH, Martinsried, Germany). In brief, 15 µg of AM proteins were solubilized with lysis buffer and subsequently proteolyzed with trypsin. Tryptic peptides were washed, eluted from the iST column, and vacuum-dried. After drying, the peptides were resuspended in solvent A (0.1% trifluoroacetic acid [TFA] in water/acetonitrile [ACN] [98:2, v/v]), and approximately 2 µg of each sample was subjected to LC–MS/MS analysis on an UltiMate 3000 RSLCnano System (Thermo Scientific, Bremen, Germany) connected online to an LTQ Orbitrap Elite (Thermo Fisher Scientific, Bremen, Germany) equipped with a pneu-Nimbus dual ion source (Phoenix S&T, Chester, PA, USA). Trapping was performed at 10 µl/min for 4 min in solvent A on a 20-mm trapping column (made in-house, 100 µm internal diameter, 5-µm beads, C18 ReproSil-HD, Dr. Maisch, Germany), and the sample was loaded on a 200-cm-long micro-pillar array column (PharmaFluidics, Ghent, Belgium) with C18 end-capped functionality mounted in the UltiMate 3000 column oven at 50 °C. For proper ionization, a fused silica PicoTip emitter (10 µm inner diameter, New Objective, Littleton, MA, USA) was connected to the µPAC™ outlet union, and a grounded connection was provided to this union. Peptides were eluted by a non-linear increase from 1 to 55% solvent B (0.1% formic acid [FA] in water/ACN [2:8, v/v]) over 137 min, first at a flow rate of 750 nl/min, then at 300 nl/min, followed by a 13-min wash reaching 99% solvent B and re-equilibration with solvent A (0.1% FA in water).

The mass spectrometer was operated in data-dependent acquisition, positive ionization mode, automatically switching between MS and MS/MS acquisition for the 20 most abundant peaks in each MS spectrum. The source voltage was 3.3 kV and the capillary temperature was 275 °C. In the LTQ Orbitrap Elite, full-scan MS spectra were acquired (*m*/*z* 300–2000, automatic gain control [AGC] target 3 × 10^6^ ions, maximum ion injection time 100 ms) with a resolution of 60,000 (at 400 *m*/*z*). The 20 most intense ions fulfilling predefined selection criteria (AGC target 5 × 10^3^ ions, maximum ion injection time 20 ms, spectrum data type: centroid, exclusion of unassigned and one positively charged precursors, and dynamic exclusion time 20 s) were then isolated in the linear ion trap and fragmented in the high-pressure cell of the ion trap. The collision-induced dissociation (CID) collision energy was set to 35%, and the polydimethylcyclosiloxane background ion at 445.120028 Da was used for internal calibration (lock mass).

### Mass spectrometric data analysis

Protein identification from the MS data was realized with the Andromeda peptide database search engine integrated into the computational proteomics platform MaxQuant (version 1.6.3.4, Max Planck Institute of Biochemistry, Planegg, Germany) [25] with default search settings including a false discovery rate set at 1% on both the peptide and the protein level. Spectra were searched against *R. prolixus* proteins (UniProt Tax ID: 13249) in the UniProt/Swiss-Prot reference database (UniProt Proteome ID: UP000015103) and the decoy database. Andromeda search parameters for protein identification specified a first search mass tolerance of 20 parts per million (ppm) and a main search tolerance of 4.5 ppm for the parental peptide. Enzyme specificity was set to C-terminal to arginine and lysine, also allowing cleavage at arginine/lysine–proline bonds with a maximum of two missed cleavages. Variable modifications were set to oxidation of methionine and acetylation of protein N-termini and fixed modification to cysteine carbamidomethylation. A minimum of two unique peptides were required for quantification. We allowed for matching between runs using a 1.5-min match time window and a 20-min alignment time window. Proteins were quantified by the MaxLFQ algorithm integrated in MaxQuant software. A minimum ratio count of two unique or razor peptides was required for quantification. Further data analysis was performed with the Perseus software (version 1.6.2.1, Max Planck Institute of Biochemistry, Planegg, Germany) after loading the protein groups file obtained previously by MaxQuant software. First, proteins identified by site and reverse database hits were removed, and label-free quantification (LFQ) values were log2 transformed to achieve normal data distribution. Data from four biological replicates of each condition were grouped as two different conditions, and proteins with less than three valid values in at least one condition were removed. Then, missing values from the other condition were imputed with values from the lower part of the normal distribution representing the detection limit. The statistical significance of changes in abundance between sample groups was calculated by a two-tailed *t*-test, with *P*-values adjusted for multiple testing by a permutation-based false discovery rate (FDR) at 5% using Perseus software. Microsoft Excel was used to calculate ratios and fold changes (FC) followed by log2 transformation. Only proteins with FC ≥ 2 and *P*-value ≤ 0.05 were considered.

### Functional classification of differentially expressed proteins (DEPs)

UniProt ID numbers from the protein list generated by Perseus were searched against UniProtKB using the Retrieve/ID mapping tool (https://www.uniprot.org/uploadlists) (accessed September 2023). This allowed us to associate UniProt accession to the corresponding protein names, Gene Ontology (GO) categories and their IDs, molecular functions, protein families, subcellular locations, biological processes, signal peptides, and VectorBase IDs. Protein classification was then performed according to GO hierarchy. Additionally, some proteins were classified based on the presence of functional domains using InterPro (https://www.ebi.ac.uk/interpro/) and Pfam (http://pfam.xfam.org/) (accessed September 2023). The subcellular location of DEPs was predicted using Euk-mPLoc 2.0 [26].

## Results

### *Trypanosoma cruzi* modulates the anterior midgut proteome of its vector

**Fig. 1:**
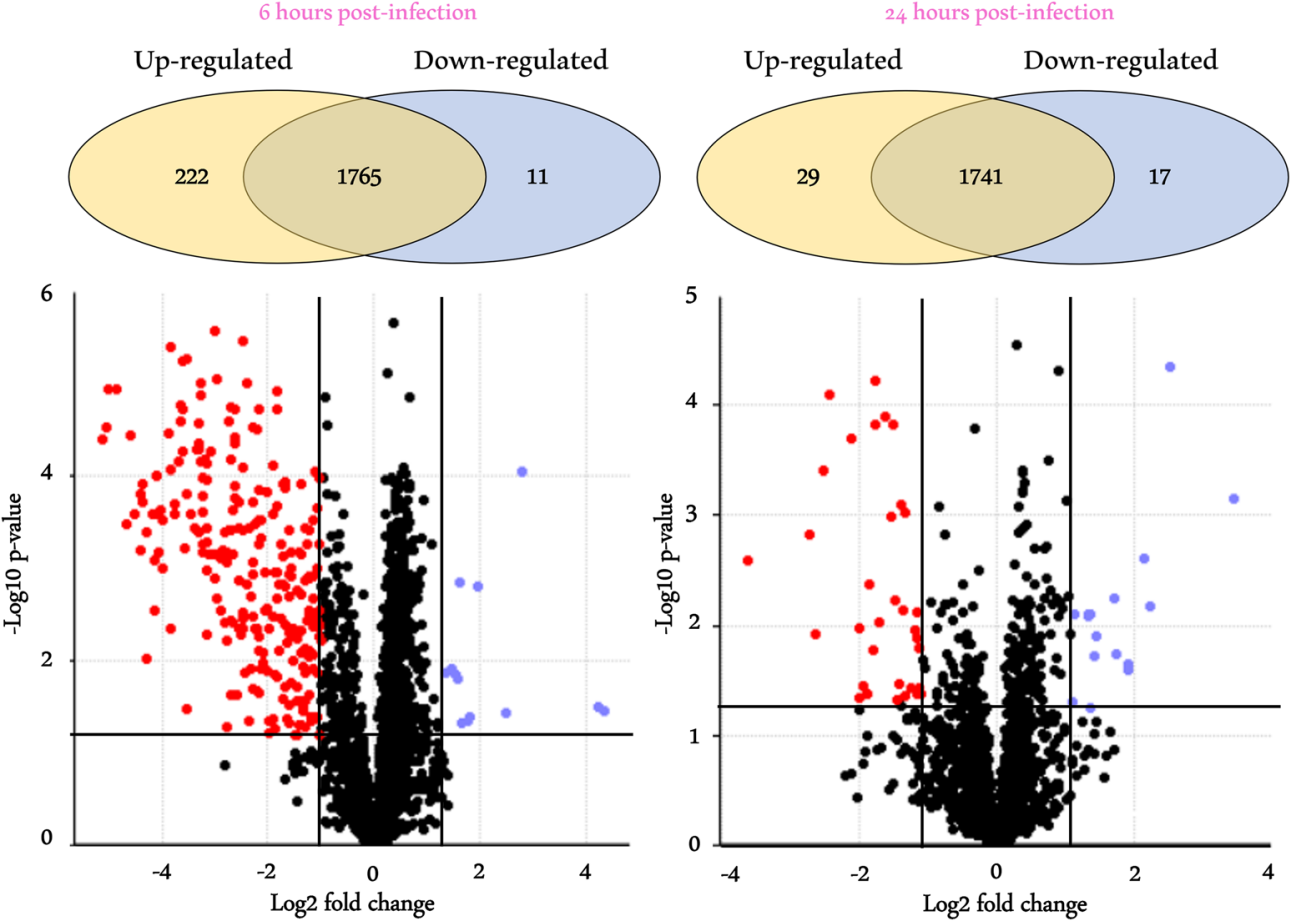
Volcano plot showing the differential protein expression between uninfected and *T. cruzi*-infected *R. prolixus* AM. Differential analysis of the AM proteome of uninfected and infected insects at 6 and 24 h post-feeding. *y*-axis: negative log10 of *P*-value; *x*-axis: log2-transformed fold change; red dots: upregulated proteins with significant *P*-value; blue dots: downregulated proteins with significant *P*-value; black dots under the significance lines: non-variable proteins. Differentially expressed proteins were determined by Student’s *t*-test (*P* ≤ 0.05) and FC ≥ 2

In this investigation, we delved into the repercussions of *T. cruzi* infection on midgut physiological dynamics in *R. prolixus*. This exploration involved a differential analysis of the AM proteome of uninfected and infected insects at and 24 h post-feeding. This comparison enabled the identification of 218 upregulated proteins at 6 hpi and 29 proteins at 24 hpi. Conversely, the presence of *T. cruzi* adversely affected the expression of 11 proteins at 6 hpi and 17 proteins at 24 hpi (Fig. 1). On the other hand, a total of 1765 and 1741 proteins exhibited no change in expression levels between uninfected and infected insects at 6 and 24 hpi, respectively.

### Functional classification of *T. cruzi*-regulated proteins

**Fig. 2:**
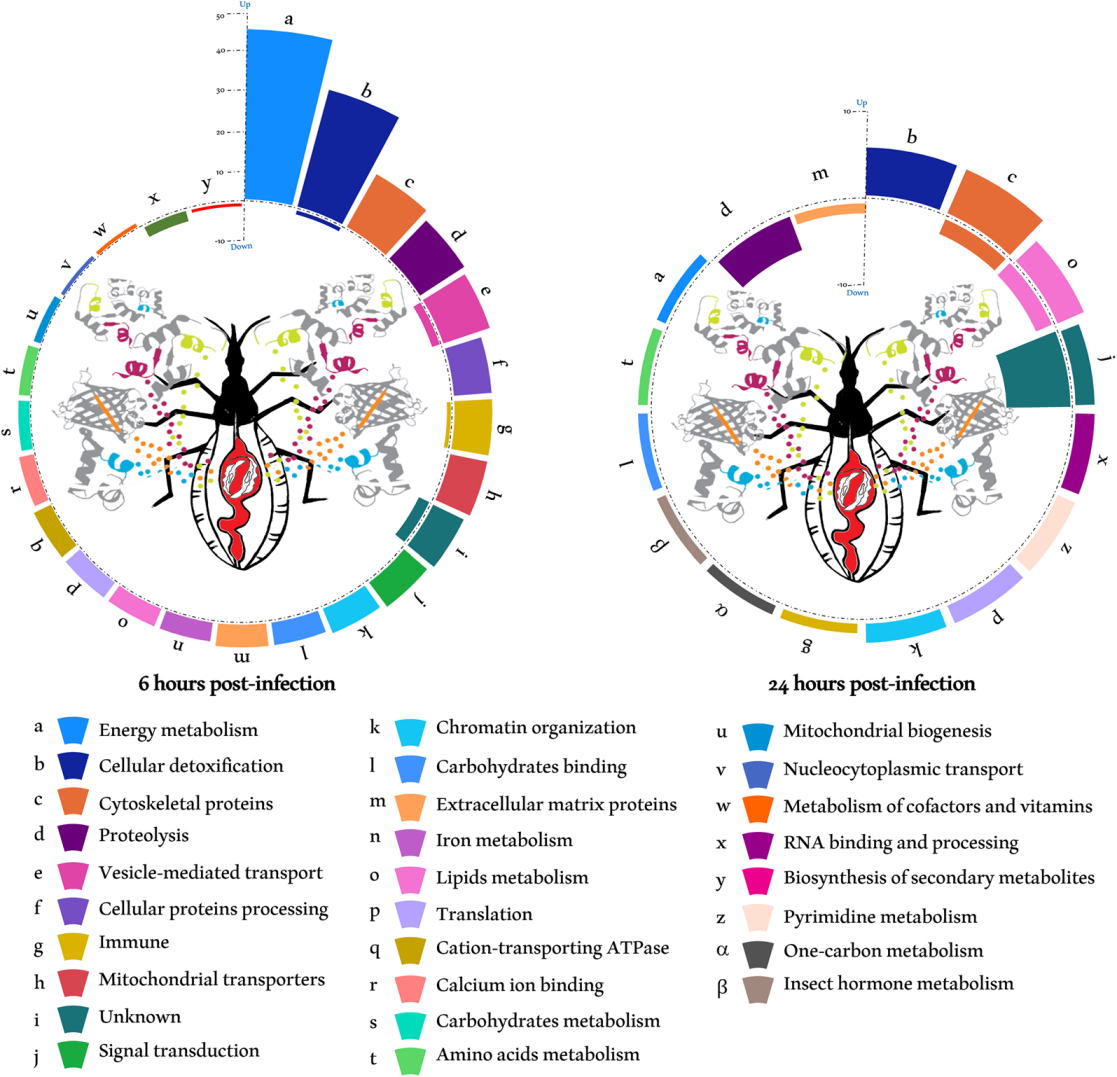
Functional classification of *T. cruzi*-regulated proteins in the AM of *R. prolixus* at 6 and 24 hpi. The differentially expressed proteins were categorized based on their functional roles in specific biological processes. This classification relied on Gene Ontology (GO) annotations as well as the presence of functional domains within the protein sequences. The bar height for each biological process represents the level of the differential expression

Classification of proteins exhibiting altered expression in the insect's digestive tract following *T. cruzi* ingestion revealed that among the 218 upregulated proteins at 6 hpi, 45 proteins are involved in cellular energy metabolism (Fig. 2). They include nine subunits of the ATP synthase complex and 36 proteins of the respiratory chain (Table S1). Several proteins of this category exhibited pronounced induction, such as the ATP synthase subunit e, whose expression was increased by 50-fold in response to the parasite (Table S1). The second major category of proteins induced by the parasite consists of 32 proteins involved in detoxification processes whose expression levels varied from twofold to 23-fold (Table S1). Notably, this category encompasses 21 cytochrome P450, three UDP-glucuronosyltransferases, one cytochrome b5 (R4G7X8), and several antioxidant enzymes, including two thioredoxins (T1H7X1 and T1HTP6), a glutathione transferase (T1HCK9 and T1IEE7), and a superoxide dismutase (R4FMI6) (Table S1).

In addition, the expression of 16 cytoskeletal proteins was affected by the parasite infection (Fig. 2), notably those of the myosin–actin–troponin–tropomyosin scaffold. Interestingly, the expression level of two of them (T1I0H5 and T1HCT9) is maintained until 24 hpi (Table S1). *Trypanosoma cruzi* also affected the expression of different proteases belonging to various families. This modulation in protein expression ranges between threefold and 24-fold. Within the affected proteases, 13 displayed the highest expression levels at 6 hpi encompassing three members of the aspartyl protease family (R4G5J4, R4FKP9, and R4FNG1), two cysteine peptidases (T1IA61 and T1HFH0), two aminopeptidases (T1HEX5 and T1HEX2), and the dipeptidase T1HMZ0 (Table S1). Furthermore, the parasite ingestion modulates the expression of a mitochondrial metalloprotease belonging to the M16 family (R4G4U9). An intriguing discovery was the significant upregulation, by a remarkable 24-fold, of the prohibitin-related membrane protease R4G8A1 in response to parasite ingestion (Table S1). This pronounced increase in protein expression raises compelling questions regarding its potential role. Hence, it might be correlated with the upregulation of two mitochondrial prohibitins (R4G4P1 and T1HZB4), whose expression increased by 12- and 13-fold, respectively, and the modulation of six intra-mitochondrial transporters (Table S1). This interconnected upregulation hints at a complex interplay with the mitochondrial proteome orchestrated by the parasite.

The parasite promoted the expression of ten immune-related proteins, including three putative DM9 repeat proteins (T1HAB7, T1HHK0, and T1I462), a putative scavenger receptor class B (R4FK91), a protein with a Sushi domain (T1ICZ1), an immunoglobulin (Ig)-like domain-containing protein (T1I7L3), an Ig I-set domain-containing protein (T1H7W0), and a leucine-rich repeat protein (T1IG52). On the other hand, the expression of lysozyme A9LN32 was downregulated fourfold by *T. cruzi* at 6 hpi.

The other most represented proteins induced during the early stage of infection are associated with vesicular transport (13 proteins) and involved in cellular protein processing and modification (10 proteins), signal transduction (eight proteins), and chromatin organization (seven proteins) (Fig. 2 and Table S1).

Six carbohydrate-binding proteins underwent substantial upregulation at 6 hpi. Specifically, two L-type lectins (R4FQE8 and T1HEJ1) show a sevenfold increase in expression. Additionally, four proteins possessing a chitin-binding domain (R4G2V8, R4G5M5, T1HFZ1, and R4G8D1) were significantly upregulated (Table S1). Noteworthy among them is a chitin-binding peritrophin (R4G5M5), displaying 14-fold induction. Furthermore, other less prevalent categories include proteins involved in lipid metabolism (five proteins), translation (five proteins), amino acids metabolism (three proteins), and iron metabolism (five proteins) comprising four ferritins (R4G4L4, T1HYY6, T1I6V1, and T1H8P8) and a transferrin (T1HAU6). Additionally, the parasite induces the expression of two alpha-glucosidases (R4G3S6 and T1I9J2) and an L-fucosidase (R4G3J4), primarily involved in carbohydrate metabolism. Among the 218 upregulated proteins observed at 6 hpi, the functions of nine remain unknown. Two of them showed pronounced regulation, with a 24-fold increase for T1HAI6 expression and 17-fold for R4G4B7.

In addition to lysozyme, the parasite negatively regulated the expression of 10 proteins at 6 hpi (Fig. 1 and Table S1). One notable example is mevalonate kinase (T1IDA7), showing 23-fold down-regulation.

At 24 hpi, the parasite presence in the vector’s AM induced the expression of various proteins involved in diverse metabolic pathways (Fig. 2 and Table S1). This includes flavin-containing monooxygenases (T1IB60, T1IB86, and T1IB95) responsible for xenobiotic detoxification, five cytoskeletal proteins, three proteins involved in lipid metabolism, two in pyrimidine synthesis, two antioxidant enzymes, and two proteins with unknown function (T1HIJ6 showing the highest fold change 24 hpi). Additionally, a juvenile hormone-binding protein (JHBP, A0A4P6D993), which plays a pivotal role in the synthesis of the key hormone for insect physiology, showed a fivefold increase in expression. In parallel, the parasite downregulated the expression of 17 proteins (Fig. 2 and Table S1), including four proteases and eight proteins with unknown function. Among the proteases whose expression is altered by the parasite at 24 hpi, we identified two carboxypeptidases (R4FLC5 and R4G7X4) and two aspartic peptidases of the A1 family (R4FNN7 and T1I914) with 11 and sixfold decrease, respectively. Interestingly, the two aspartic peptidases are different from those induced by the parasite 6 hpi.

### Alteration of mitochondrial homeostasis in the anterior midgut epithelial cells by *T. cruzi*

**Fig. 3:**
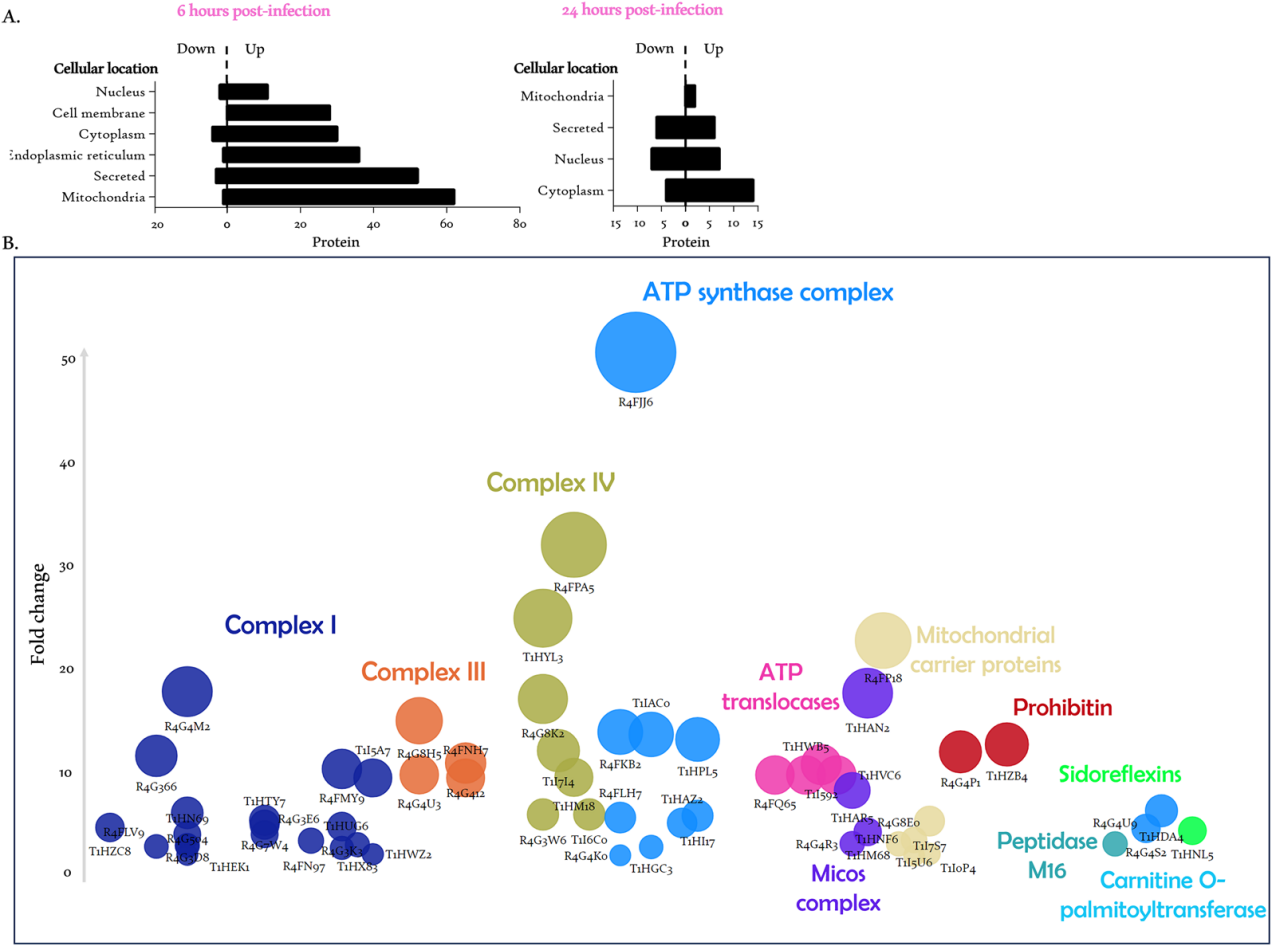
Mitochondrial physiological changes in *R. prolixus* AM in response to *T. cruzi* infection. **A** Subcellular location of DEPs at 6 h and 24 hpi by *T. cruzi*. **B** Bubble chart illustrating upregulated proteins 6 hpi associated with various mitochondrial functions. Proteins are clustered based on their respective mitochondrial functions, each represented by a distinct color. The bubble size is correlated to its FC following infection

The investigation of the subcellular localization of DEPs revealed that among the 218 proteins upregulated by the parasite in the early hours pi, 62 were mitochondrial (Fig. 3A). Grouping these proteins based on their function within the mitochondria has distinctly identified protein complexes involved in its key processes. Specifically, the parasite significantly upregulated the expression of 20 proteins contributing to the formation of complex I (NADH dehydrogenase), the first enzyme of the respiratory chain (Fig. 3B). This modulation shed light on the parasite's impact on mitochondrial function and energy metabolism. Indeed, the induction of complex I expression is correlated with the induction of four proteins (R4G412, R4G8H5, R4G4U3, and R4FNH7) involved in the assembly of complex III (cytochrome bc1) and six proteins (R4G3W6, T1HYL3, R4G8K2, T1HM18, T1I7I4, T1I6C0, and R4FPA5) from complex IV (cytochrome *c* oxidase) within the respiratory chain. Moreover, this orchestrated upregulation extends to nine proteins of the last enzyme of the respiratory chain, the adenosine triphosphate (ATP) synthase complex, with substantial inductions reaching up to 50-fold, particularly notable for the subunit e (R4FJJ6). In addition, the parasite-induced expression of various mitochondrial transporters, such as three ATP translocases (R4FQ65, T1HWB5, and T1HVC6), two sideroflexins (T1HDA4 and R4G4S2), a porin (R4G4R3), a carnitine palmitoyltransferase (T1HNL5), and other mitochondrial carrier proteins with a 23-fold induction for R4FP18. On the other hand, a mitochondrial intermembrane space protein TIM13 (R4G4D7) showed an 11-fold downregulation at 6 hpi. This sophisticated modulation of protein expression includes an intriguing induction of multifaceted proteins, such as two prohibitins (R4G4P1 and T1HZB4) and three proteins (T1HM68, T1HAN2, and T1HAR5) from the mitochondrial contact site and cristae organizing system (MICOS) complex, known for their pleiotropic roles.

## Discussion

Interactions between pathogens and their vectors occur throughout their intra-vectorial cycle and up to their transmission to the next host. Unlike *Trypanosoma rangeli*, which is pathogenic to its vector and can invade the hemolymph, *T. cruzi* undergoes its entire vectorial life cycle within the vector’s gut [12]. This implies constant contact with the vector's digestive tissues, microbiota, and various compounds secreted during the digestive process of the blood meal. Indeed, hematophagy induces several changes in the gut environment of the triatomine, especially in the AM, such as changes in temperature, osmolarity, pH, and oxidative stress [4, 8]. These changes make this organ an inhospitable environment for the development of *T. cruzi*. In fact, around 90% of the parasites are lysed in the AM, possibly by a factor released by the insect during digestion [6, 7]. Therefore, the parasite must fine-tune its metabolic pathways, energy utilization, and resource allocation to align with the new host environment. For instance, to generate its energy, the parasite must utilize amino acids abundantly available in the digestive tract instead of glucose [18]. Furthermore, although *T. cruzi* is considered sub-pathogenic for triatomines, it has been observed that its presence in the digestive tract alters several behavioral and physiological traits of the vector [20, 27], without yet understanding the molecular basis underlying these alterations. For instance, it has been demonstrated that *R. prolixus* is sensitive to *T. cruzi* infection and its presence triggers a systemic immune response [10, 13].

Interactions between triatomines and trypanosomes are intricate, and many questions persist regarding the nuanced molecular interplay between them. In this study, we assessed the impact of *T. cruzi* infection on *R. prolixus* by analyzing the protein expression profile within the AM at 6 and 24 hpi using quantitative proteomics. Shortly after ingestion, the protozoan profoundly influences the dynamics of this tissue, significantly increasing the expression of 218 proteins and downregulating 11 proteins (Fig. 1). At 24 hpi, the impact of the parasite's presence on protein expression in the AM is less pronounced, characterized by the upregulation of 29 proteins and the downregulation of 17 proteins. The temporal disparity in the expression profile could potentially be attributed to several factors. Firstly, it may result from parasite migration towards the PM [6]. Additionally, the decline in the systemic immune response initially triggered by the parasite lysis could also contribute to this temporal disparity. However, the influence of the microbiota and physicochemical factors (e.g., temperature) cannot be discarded.

While it has previously been demonstrated that *T. cruzi* induces a transcriptional response in *T. infestans* [22] and *R. neglectus* [23], this study is the first to elucidate the effect of *T. cruzi* on *R. prolixus* AM at the protein level.

The DEPs identified in this study are engaged in a wide array of cellular functions, many of which are pivotal due to their instrumental roles in cellular metabolism and homeostasis (Fig. 2 and Table S1). An in-depth analysis of these proteins modulated by the parasite at 6 hpi revealed a predominance of mitochondrial proteins (Fig. 3). Indeed, the parasite significantly affected the expression of 62 mitochondrial proteins, thereby altering the proteomic landscape of this organelle.

Mitochondria exhibit remarkable functional versatility. In addition to their role in cellular energy production, mitochondria also play a crucial role in regulating innate immune responses against infections [28, 29]. Therefore, it is well established that maintaining mitochondrial homeostasis in the midgut significantly impacts an insect’s fitness and resistance against pathogens [30, 31]. Beyond the established role of mitochondria in the immune response, recent research has shed light on the intricate involvement of the respiratory chain—a fundamental complex crucial for cellular energy production, the generation of reactive oxygen species (ROS), and the regulation of apoptosis in immunity [32]. Twenty subunits of the mitochondrial complex I were modulated by the presence of the parasite at 6 hpi (Fig. 3). Mitochondrial complex I is the largest enzyme complex in the oxidative phosphorylation pathway and the entry point for electrons into the respiratory chain. It is also a major generator of intracellular ROS, which are considered important signaling molecules with intricate physiological effects [28]. Hence, murine studies have revealed the pivotal function of GRIM-19 (NADH dehydrogenase 1 alpha subcomplex subunit 13) in orchestrating immune responses [32]. In addition, a 200-fold upregulation of NADH dehydrogenase subunit 5 transcript was observed in human intestinal epithelial cells infected with the non-invasive enteric *Vibrio cholerae* [33]. Interestingly, this upregulation was related to both motility and adherence of the bacterium to intestinal epithelial cells. A mitochondrial glycerol-3-phosphate dehydrogenase (T1I592, mGPDH) showed an 11-fold increase at 6 hpi. mGPDH is a very important enzyme at the junction of glycolysis, oxidative phosphorylation, and fatty acid metabolism [34]. In addition, mGPDH activity has been correlated with ROS production [35]. Given that proteins involved in the composition of the respiratory chain complexes, including NADH dehydrogenase 1 alpha subcomplex subunit 13 (R4FLV9) and subunit 5 (R4FMY9), are significantly upregulated (threefold and 10-fold, respectively) in the AM shortly after the establishment of the parasite (Fig. 3 and Table S1), it suggests that the parasite triggers a complex signaling cascade which is related to infection. The intricate regulation of mitochondrial activity within the midgut relies predominantly on the insulin/insulin-like growth factor signaling (IIS) cascade [36]. While the insulin receptor and its conserved intracellular signaling pathway have been outlined in *R. prolixus* [37], neither the insulin receptor nor the array of transcriptional factors and actors within this pathway show a significant correlation with the alteration of mitochondrial dynamics induced by the trypanosome. This suggests the involvement of an alternative pathway governing mitochondrial dynamics in the midgut.

Interestingly, we identified a noteworthy upregulation of two mitochondrial prohibitins, R4G4P1 and T1HZB4, orthologs to the mitochondrial prohibitin complex subunit PHB1 and PHB2, respectively. These proteins form a distinctive ring-like macromolecular structure within the inner mitochondrial membrane, critically contributing to a diverse array of crucial cellular processes, notably including mitochondrial biogenesis [38, 39]. Concomitant with the overexpression of these prohibitins induced by the parasite, a substantial induction of the putative prohibitin-related membrane protease (R4G8A1) was observed. It has been demonstrated that the absence of PHB1 leads to an instability of mitochondrial-encoded subunits of the respiratory chain [40]. Furthermore, in mammals, the downregulation of PHB1 results in reduced expression levels of specific complex IV subunits encoded by both mitochondrial and nuclear DNAs, consequently associated with a decline in enzymatic activity [39, 41]. Additional research revealed that silencing of either PHB1 or PHB2 causes a reduction in the activity of other respiratory chain complexes [42]. Moreover, the overexpression of PHB1 has been shown to enhance complex I-dependent mitochondrial respiration, providing protection against oxidative stress and promoting ATP generation [43, 44]. Modulation of these two scaffold proteins of the inner mitochondrial membrane could be associated with the observed MICOS complex (MIC10, 13, and 60) upregulation. Both prohibitins and MICOS are involved in the structure of the cristae, which house the respiratory chain complex [45]. In addition, the regulation of two mitochondrial translocases of the outer membrane Tom40 (R4G4R3) and the inner membrane Tim13 (R4G4D7) confirms the cross-talk between the respiratory chain, the MICOS complex, and the inner and outer mitochondrial membranes [46]. Mitochondrial dynamic and motility are related [47], which brings into play the important number of variable cytoskeletal proteins observed 6 hpi (Fig. 2 and Table S1). Taken together, the compelling arguments substantiated by our results strongly support the conclusion that the modulation of respiratory chain protein expression and the potential surge in mitochondrial activity are mediated through the large complex of prohibitins. Moreover, the increase in the abundance of the respiratory chain proteins agrees with ROS production and consequently with a marked increase in the expression of various antioxidant enzymes, including superoxide dismutase and thioredoxin, which was observed following the infection. Similarly, mirroring the pattern observed in respiratory chain proteins, the expression levels of these antioxidant enzymes returned to a steady state, showing no significant difference between infected and control insects after 24 hpi.

Twenty-one isoforms of P450 cytochromes (Table S1) exhibited a prominent and swift induction in response to *T. cruzi* (Table S1). Nineteen isoforms belong to clan CYP3 and two isoforms to clan CYT4 [48]. It has been suggested that proteins within a clan share common functions and CYP3 has the largest gene expansion of P450 cytochromes in *R. prolixus* [49], which correlates with the important number of variable P450 isoforms observed upon infection in this work. Proteins of these two clans were proposed to have an important detoxification role and their expansion was linked to insecticide resistance [50]. Additionally, cytochromes P450 hold significance in fundamental biological processes, encompassing growth, development, feeding, reproduction, and oxidative stress [51–53]. Notably, in triatomines, they have been recognized for their contribution to insecticide resistance [48, 54–56] and hydrocarbon formation [57]. Also, in *Anopheles gambiae*, cytochromes P450 seem to have immune implications as their expression is induced in response to microbial challenge [58, 59]. Remarkably, in the context of malaria infection, differential expression patterns of cytochromes P450 have been observed throughout various phases of midgut invasion by *Plasmodium* [60]. The consistent observation of differential expression of cytochromes P450 within the initial hours of *T. cruzi* invasion and the absence of the modulation of the complement and important detoxification enzymes esterases and glutathione s-transferases argue for the potential role of cytochromes P450 in the host's response to the parasite.

Among the components of the midgut epithelium's response to parasite infection, a notable upregulation of three DM9 domain-containing proteins has been observed. These proteins are recognized for their role as pattern recognition receptors (PRRs) with a broad microbial recognition spectrum [61]. An illustrative instance is the *An. gambiae* PRS1, which functions as an agonist in the development of the *Plasmodium* parasite within both midgut and salivary gland tissues [62]. Exploring the function of these proteins in *R. prolixus* could potentially unveil novel insights into the molecular cross-talk with *T. cruzi.* Moreover, our investigation revealed the presence of an Ig I-set domain-containing protein (T1H7W0). Proteins featuring this domain, such as hemolin [63, 64], play a crucial role in various functions, including the opsonization of pathogens by acting as pattern recognition receptors [65, 66]. Notably, we previously identified T1HCN4 to be expressed in the hemolymph of *R. prolixus* following both blood ingestion and *T. cruzi* challenge [13]. Additionally, *T. cruzi* is observed to upregulate the expression of the tumor necrosis factor (TNF) superfamily ligand ortholog (T1I618). In invertebrates, Eiger has the distinction of being the first and unique TNF ligand characterized in *Drosophila melanogaster* [67]. Eiger has been extensively validated for its multifaceted roles, including its capacity to initiate the c-Jun N-terminal kinase (JNK) pathway, thereby inducing programmed cell death [68]. Furthermore, it is a key player in the innate immune response [69], with a particular emphasis on defense against extracellular pathogens [70]. While the involvement of TNF ligand in triatomines has yet to be explored, it is conceivable that it may act as a trigger for a cascade leading to metabolic reprogramming in the midgut. This signaling pathway could potentially play a role in the downregulation of lysozyme. Intriguingly, although blood feeding induced the expression of lysozyme (A9LN32) in the AM 6 h post-feeding in *R. prolixus* [71], the parasite exerts the opposite response at 6 hpi in line with the observed early reduction of lysozyme transcript in salivary glands upon *T. cruzi* infection [23]. Interestingly, at 24 hpi, no significant difference was observed in its expression between the two compared conditions which confirms the observed temporal regulation of lysozyme transcript [23].

The establishment of the parasite intricately influences the expression of multiple proteases belonging to distinct families. At 6 hpi, three members of the aspartic protease family A1 exhibit a significant upregulation, suggesting an active response to the presence of the parasite. However, intriguingly, one isoform within this family (R4FNN7) displays an 11-fold downregulation at 24 h, signifying a dynamic regulation of these enzymes in response to the evolving infection. This isoform was sevenfold downregulated by the blood meal 6 h post-feeding [71]. Moreover, the three isoforms belong to different branches in the phylogenetic trees of protease family A1 [72]. In addition to their physiological role within the AM [73], these proteases seem to extend their influence on the interaction with the parasite, potentially mediating crucial processes. Moreover, previous investigations have highlighted an augmentation in their enzymatic activity following *T. cruzi* infection of *R. prolixus* [74], reinforcing their significance in the intricate dynamics of host–parasite interplay and warranting further in-depth exploration.

Remarkably, at 24 h following the establishment of the parasite, there is a notable induction in the expression of the JHBP A0A4P6D993. Intriguingly, the expression pattern of this protein within the AM mirrors the expression of the hemolymph JHBP (R4FK69). These transporters play a pivotal role in transporting JH, which governs a multitude of physiological functions critical for insect homeostasis [75–77]. Notably, research on *D. melanogaster* has demonstrated that JH exerts an antagonistic effect on the induction of antimicrobial peptide expression following an immune challenge [78]. Intriguingly, recent studies have highlighted the essential role of JHBP in *Aedes aegypti*, revealing its significance in regulating innate immune response and hemocyte development [79]. Consequently, it is conceivable that JHBP in *R. prolixus* harbors immune implications, influencing humoral and cellular responses against *T. cruzi* in both the midgut and the hemolymph. Delving deeper into the functional aspects of JHBP in immune competence could shed light on the endocrinological regulation of the immune response in triatomines, potentially unraveling novel facets of their immune regulatory mechanisms.

In this work, we meticulously delineated the events induced by *T. cruzi* in its vector during the initial hours of its vectorial cycle within the AM. This exploration shed light on fundamental aspects of host–pathogen interactions. Although we now understand that the parasite alters the cellular physiology of the digestive tube, in particular by modifying mitochondrial dynamics, it remains unclear whether this reprogramming is responsible for the parasite's lysis, possibly orchestrated by an overproduction of ROS by the respiratory chain. Alternatively, it could simply be a consequence of the parasite lysis, accompanied by the release of cellular content that may induce damage to the intestinal epithelium, subsequently triggering an apoptotic response involving the deregulation of mitochondrial dynamics. Further in-depth exploration of the mechanisms underpinning the molecular dialogue between *T. cruzi* and its vector is of paramount importance. Such insights could pave the way to effectively impede the parasite's development and, in turn, curtail its transmission to the human host.

## Supplementary Information

Supplementary file 1.

## Data Availability

The mass spectrometry proteomics data have been deposited to the ProteomeXchange Consortium via the PRIDE [80] partner repository with the dataset identifier PXD051212.
